# Molecular Biology of Atherosclerotic Ischemic Strokes

**DOI:** 10.3390/ijms21249372

**Published:** 2020-12-09

**Authors:** Antonino Tuttolomondo, Maria Grazia Puleo, Maria Chiara Velardo, Francesca Corpora, Mario Daidone, Antonio Pinto

**Affiliations:** Department of Health Promotion, Maternal and Infant Care, Internal Medicine and Medical Specialties, “G. D’Alessandro”, University of Palermo, Piazza delle Cliniche n.2, 90127 Palermo, Italy; dott.ssamgpuleo@gmail.com (M.G.P.); mariachiara.velardo@libero.it (M.C.V.); francesca.corpora@gmail.com (F.C.); mariodaidone@gmail.com (M.D.); antonio.pinto@unipa.it (A.P.)

**Keywords:** ischemic stroke, neuroinflammation, atherosclerosis, microglia, NLRP3 inflammasome, DKK-3, Dectin-1, MKEY, microRNAs, CD200-CD200R, AF, BBB

## Abstract

Among the causes of global death and disability, ischemic stroke (also known as cerebral ischemia) plays a pivotal role, by determining the highest number of worldwide mortality, behind cardiomyopathies, affecting 30 million people. The etiopathogenetic burden of a cerebrovascular accident could be brain ischemia (~80%) or intracranial hemorrhage (~20%). The most common site when ischemia occurs is the one is perfused by middle cerebral arteries. Worse prognosis and disablement consequent to brain damage occur in elderly patients or affected by neurological impairment, hypertension, dyslipidemia, and diabetes. Since, in the coming years, estimates predict an exponential increase of people who have diabetes, the disease mentioned above constitutes together with stroke a severe social and economic burden. In diabetic patients after an ischemic stroke, an exorbitant activation of inflammatory molecular pathways and ongoing inflammation is responsible for more severe brain injury and impairment, promoting the advancement of ischemic stroke and diabetes. Considering that the ominous prognosis of ischemic brain damage could by partially clarified by way of already known risk factors the auspice would be modifying poor outcome in the post-stroke phase detecting novel biomolecules associated with poor prognosis and targeting them for revolutionary therapeutic strategies.

## 1. Introduction

Stroke is termed as a sudden onset of focal or global symptoms and clinical signs, due to an abrupt occlusion or rupture of a blood cerebral vessel which determines consequential brain suffering through cerebral hypoperfusion or resultant compression by hemorrhage.

Clinical manifestations persist more than 24 h, and neuroimaging techniques like CT or MRI could additionally uphold diagnosis.

The high incidence worldwide of stroke, associated with elevated mortality and morbidity, makes this chronic problem a plague of the whole human society.

Every year 15 million people are afflicted by a cerebrovascular accident, and epidemiological data show that one-third of them go to death, and another one-third is affected by a severe physical and psychological disability.

Several studies suggested a correlation between the incidence of stroke and ethnic factors; Hispanic and Black people have a significant risk to be suffering from the invalidating pathology above-mentioned compared to Caucasians in the United States [1].

Therefore, identifying new etiopathogenetic factors of an ischemic cerebrovascular event is necessary with the intention to find new therapeutic regimens capable of affecting prognosis and quality of life of patients.

There are two primary physiopathological dichotomous substrates: Ischemia and haemorrhage.

In the first circumstance, a discrepancy between intracellular oxygen requirement and brain inflow occurs due to an occlusion of a brain blood vessel by a clot (thrombus or embolus). In contrast, in the second case, the hematic extravasation consequent to vascular rupture provokes compression of adjacent brain parenchyma. Brain ischemia constitutes the most common physiopathological entity of a cerebrovascular lesion.

Five distinct subcategories of stroke are described by TOAST classification [2]. They could be large-artery atherosclerosis (45% of the total of events), cardioembolism (15–30%), systemic hypoperfusion, penetrating artery disease, carotid dissection, hypercoagulability of genetic syndromes or with an undetermined etiology.

Several processes like atherosclerosis, endothelial dysfunction and neuroinflammation subtend the pathophysiology of ischemic stroke.

A substantial contribution to the etiopathogenesis of ischemic damage is made by the immune system which not only mediates inflammatory cascade but also determines an immune suppression, due to the effects of the vegetative nervous system, overactivated by cerebral lesion, on the lymphoid system.

Therefore, increased susceptibility to elapsing infections takes place, and it provokes a further increase of chance of mortality and morbidity after a cerebrovascular accident [3,4].

Iadecola and Anrather have drawn up a list of mediators implicated in all phases of neuroinflammation in ischemic stroke, from the beginning to the resolution. The onset of neuroinflammation after brain injury is mediated by mediators like “TNF-α, IL1α, IL1β, CXCL7, CCL5, CXCL4, CX3CL1, adhesion molecules, proteases, prostanoids, and leukotrienes”; “IL-1, IL-10, TNF-α, IL- 6, IL-20, IL-17”, and “NADPH oxidase, CXCL8, iNOS, and COX-2” are involved in the phase of boosting of this process and other molecules including “TGF-β, IL-17, IL-10, and IL-23” mediate its termination [5]. Other molecules such as NLRP3 inflammasome, DKK-3, Dectin-1, MKEY, and microRNAs are implicated in neuroinflammation so ongoing, and future researches about the intricate mechanism related could lead to pioneering therapeutic interventions that could considerably modify the natural course of this invalidating condition.

The pathobiology of stroke is utterly characterized, besides the activation of innate immunity which exacerbates brain harm through the release of inflammatory cues, by the co-participation of the adaptative immune system, elicited by massive exposure to several antigens, due to the blood–brain barrier (BBB).

In this review, we take into consideration the noxious and the curative properties of some mediators of neuroinflammation, focusing on the role of the innate and adaptative immune system and the contribution made by atrial fibrillation. Besides, we investigate the relationship between neuroinflammation and BBB dysfunction and miRNAs that plays a pivotal role in the pathogenesis of stroke, representing potentially promising therapeutic targets. We have chosen to deepen some biomolecules and related pathways which are described in very recent researches in literature and whose modulation probably could circumscribe the damaged area after a stroke, preventing the ruinous effects of inflammation on brain lesion.

## 2. The Role of the Innate Immune System in Neuroinflammation

As many evidences have demonstrated, neuroinflammation could be defined as an inflammatory response to insults derived from several pathogenic noxae related to different pathologies such as ischemic stroke [6,7]. In the latter case, neuronal death is responsible for the initiation of an inflammatory cascade piloted by chemokines, cytokines, and ROS.

Furthermore, various cell types which are involved in this process and are members of the innate immune system shall be liable of necrosis and apoptosis of cerebral cells, resulting in a further increase of brain lesion size and a more severe cognitive impairment and disability [8,9,10].

Plenty of reviews in worldwide literature treat about general aspects the pathogenetic contribution of several inflammatory cytokines and the involvement of various cell types such as neutrophils, microglia, and astrocytes in neuroinflammation, after an ischemic stroke.

Consequently, we preferred to explore other recently in-depth biomolecular pathways, which modulation could be established future opportunities for innovative strategies of treatment (Table 1).

### 2.1. NLRP3 Inflammasome

Several diseases such as autoinflammatory pathologies, diabetes, and stroke share the pathogenetic mechanism of inflammation, mediated by many molecules like cytokines, some of which are characterized by pro-inflammatory features. Between the latter, the interleukin 1β plays a pivotal part and, as many studies have illustrated, it can trigger and modulate the drop-down pathways of inflammation, through the interaction with inflammasome [23,24].

In the release of cytokines get involved different cellular sensing elements of the innate immune system, many of them belonged to the family of NOD-like receptor (nucleotide-binding oligomerization domain-like receptor), also called NLR. The latter distinguish themselves for the presence of the domain NACHT. It consists of the acronym of its most significant proteic components like a protein that inhibits neuronal apoptosis (NAIP), an activator of the transcription of class 2 MCH (C2TA), heterokaryon incompatibility, and the protein one telomerase-associated (TP1) [25].

A C-terminal leucine-rich repeat (LRR) and pyrin domains (PYD) or N-terminal caspase recruitment (CARD) enwrap the core of NATCH [26,27]. Different researches focused on NAPL3 also termed cryopyrin or NLRP3 (NLR family pyrin domain-containing 3) inflammasome, a member of the NOD-like receptor family codified by the gene NLRP3 [25]. It is indeed one of the main contributors to the process of neuroinflammation and the consequent brain damage in patients who have type 2 diabetes and cerebrovascular accident [28,29]. Moreover, the downregulation of NLRP3 could represent a powerful therapeutic approach in both the subtype of subjects aforementioned [30,31].

A sensor made up of three different domains of NLRP3 (PYD domain-containing protein, LRR and NACHT), an adaptor (ASC; also known as PYCARD) and an effector (caspase 1, a cysteine-dependent aspartate-directed protease 1) assembling themselves generate the structure of NLRP3 inflammasome.

In the event of an infection by pathogenic exogenous micro-organisms or after an intracellular injury, many distress signals such as uric acid and even hexokinase cause the activation of detriment-associated pathways. NLRP3 inflammasome has the capacity of recognizing these warnings, activating caspase 1 thanks to a previous interaction between ASC and pro-caspase 1 and having a significant impact on inflammatory mediators like IL-18 and IL-1β [32,33].

**Figure 1:**
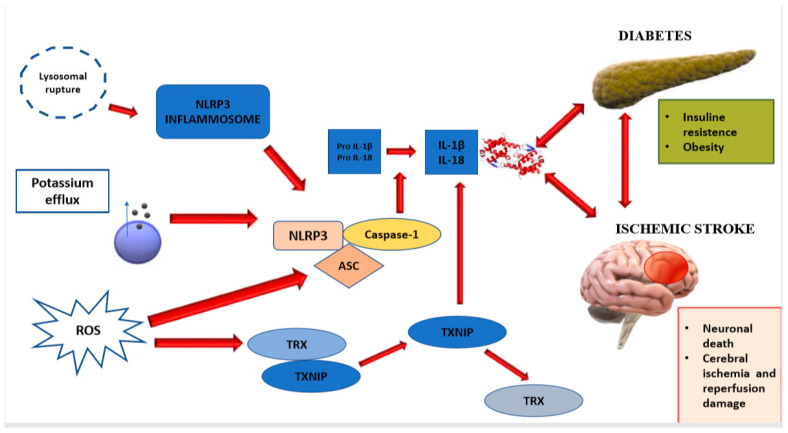
The NLRP3 inflammasome plays a fundamental part in the process of neuroinflammation consequent to a cerebrovascular accident, particularly in diabetic patients in which enhances the progression of metabolic disorder. The figure above illustrates three hypothetic etiopathogenetic factors such as lysosomal rupture, ROS and cellular potassium efflux, which are co-responsible of the activation of caspase 1, triggering the release of mediators of inflammation like cytokines IL-18 and IL-1β. In the macrophages of adipose tissue, the activation of NLRP3 inflammasome is determined by the release of DNA and the production of ROS in mitochondria, through the desegregation of TRX-TXNIP complex. After its triggering, NLRP3 inflammasome provokes the cleavage of pro-IL-1β into IL1-1β, the active form, and the release of IL-18. These cytokines phosphorylate IRS-1, as known as the insulin receptor substrate-1, worsening insulin- resistance and causing neuronal death.

It is therefore intuitive that NLRP3 inflammasome may play a pivotal role in neuroinflammation after a cerebrovascular accident, provoking the apoptosis of glial cells and neurons. In these cells, oxidative stress and endoplasmic reticulum stress is exacerbated by the release of inflammatory molecules and the activation of IL-1R signaling-IRS-1 pathway and the involvement of NLRP3 and NLRP1 inflammasome. The significant increase in the production of ROS and the continuing of NLRP3 activation aggravates cerebral damage and impairment, boosting edema and atherosclerosis processes [34,35]. This inflammasome is responsible for elevated levels of TNF-α (tumor necrosis-factor-α), a cytokine which plays a crucial role in insulin insensitivity and endothelial dysfunction [36]. Moreover, cells belonging to the innate immune system, especially microglia and macrophages, release high concentrations of IL-1β. The molecule above-mentioned worsens cerebral disablement after an ischemic stroke in diabetic patients and aggravates insulin-resistance when the comorbidity of severe obesity is present [11,12] (Figure 1).

Considering the physiopathologic implications of inflammasomes in neuroinflammation, a process responsible for poor prognosis, the therapeutic use of mediators involved in these pathways could be advantageous.

It has been shown that glyburide, sinomenine, MCC950, and other molecules reduce insulin resistance, delay advancement of ischemic brain injury, decreasing the extent and improve neurologic loss and clinical consequences.

Besides, recent evidence demonstrates that the inhibition of VDUP1 which is the acronym of vitamin D3up-regulated protein 1 (also termed TXNIP, thioredoxin-interacting protein), a signal molecule inducing the activation of NLRP3 inflammasome [37], improves brain damage. It represents a further confirmation that the crosstalk between NLRP3 inflammasome and VDUP1 can be considered a pathogenetic mechanism of ischemic stroke [38].

### 2.2. DICKKOPFF-3 (DKK-3)

Dkk-3, the acronym of Dickkopf-3 (Dkk-3), is a member of the family DKK. It is a protein which controls Wnt/β-catenin signaling pathway, decides cellular differentiation during embryogenesis [39,40] and conditions metabolism of vascular cells, decreeing their proliferative and survival fate [41]. Besides, it could also have a significant impact on the advancement of atherosclerotic burden, as recent studies established [13,42].

When Dkk-3 is overexpressed, the inhibiting action on the signaling pathway mentioned above [39,40,43], could power atherosclerosis and injuries related worsening prognosis [13] through the accretion of plaques inside the vascular lumen.

Dkk-3, indeed, regulates the angiogenetic process repressing the growth of endothelial cells determining low expression levels of VEGF (vascular endothelial growth factor) [40,44]. In contrast, a study of mice with a deficit of Apo-E shown that the underexpression of Dkk-3 could restrict the extension of atherosclerotic lesions and ameliorate the stability of plaques [13].

On the other hand, since it is an essential regulator of cell fate determination [14,45], Dkk3 was also mentioned to have a protective function against atherosclerosis. It is involved in the process of reconstitution of the endothelium in both phases of migration and repair. It also prevents the accretion of a new innermost layer of vessels in the presence of an atherosclerotic lesion, after triggering JNK pathway. In a study in knock-out mice for the gene DKK-3 with a subsequently failed expression of this protein, a significant endothelial dysfunction has been reported, evidenced by an inversely proportional relationship between the middle-intimate layer of the carotid artery and plasmatic levels of DKK-3 [31]. Given these assumptions, the goal could be represented by keeping plasmatic and tissue of the protein mentioned above within a well-defined range in physiologic conditions [14].

The study CATIS, a large-sample multicentric trial [46], demonstrated worse clinical outcomes in patients affected by an ischemic stroke, whether in the presence of high levels either of low levels of DKK-3. These findings were corroborated further by subgroup analyses, and they suggested that the relationship between DKK-3 and morbidity and mortality for cardiovascular events after an ischemic stroke follows a U-shaped trend because both the excessive and the deficient expressions predispose poor clinical consequences.

### 2.3. DECTIN-1/SYK

Several features and mechanisms influence the physiopathology of ischemic stroke. It is a well-established finding that, among all of them, neuroinflammation has greater importance in the determining the advancement of brain injury [5,30] through microglia, neuronal, and endothelial cells and other cell types belonging to the innate immune system [5].

The better prognosis observed in patients affected by an acute cerebrovascular event, after the inhibition of inflammatory pathways, represents a further finding that confirms the previous assessment [47,48].

One of these inflammatory pathways, which plays a pivotal role in neuroinflammation, is mediated by Dectin-1 and Syk, a protein with tyrosine kinase activity mainly expressed in microglia. Many pro-inflammatory signals and DAMPs, which constitutes the acronym of danger-associated molecular patterns, are identified by dendritic cells, especially some of them characterized by the expression of C-type lectin-1 (Dectin-1), thanks to the interaction with Syk [49,50,51]. In case of an inflammatory process, a higher expression of Dectin-1, a transmembrane receptor of II type, could be observed in several cell types such as T lymphocytes, dendritic cells, monocytes, neutrophil granulocytes, and cells of the epithelium [52,53,54,55].

Under physiological conditions, a low concentration of the molecule above-mentioned can be found in cerebral parenchyma; instead, it is considerably expressed when a brain injury occurs as a defensive response against damage stimuli [56,57]. Besides, it has been shown that it has an impact on the activation of inflammasome NLRP3 after the interaction with β-glucan particles [58,59,60], the subsequent augmented synthesis of ROS, and it is involved in the increased release of interleukine β [58,61,62]. The inhibition of Dectin-1 could ameliorate spinal cord injury because it antagonizes the harmful macrophagic activity, impeding axonal damage and demyelination of nerve fibers and promotes the process of neurogenesis [63,64].

**Figure 2:**
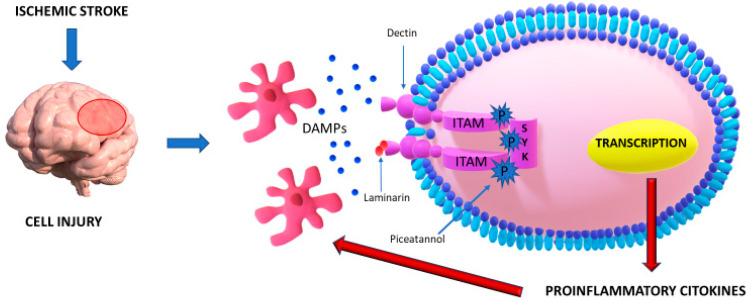
Dectin-1, also known as Dendritic cell-associated C-type lectin-1, a receptor activated by the interaction with DAMPs (damage-associated molecular patterns), is responsible for an innate immune response when brain damage such cerebral ischemia occurs. The crosstalk between Dectin-1 and DAMPs determines phosphorylation of ITAM and, subsequently, of Syk, a kinase which mediates a cascade of neuroinflammation and the release of several cytokines. Some antagonist molecules of Dectin-1 like Syk inhibitor piceatannol and laminarin can underregulate this process, reducing the detrimental effects of neuroinflammation on brain damage after a stroke.

The immunoreceptor hemITAMs, an incomplete ITAMs, is common to different types of C-type lectin receptors, like C-type lectin-like receptor 2 (also known as CLEC2), C-type lectin domain-containing 9A (which acronym is CLEC9A) and Dectin-1 and it plays a crucial part in the interaction with Syk [65]. This protein could be expressed in cells belonged to the hematopoietic system, but it can also be found in other cell types [66,67]. It constitutes one of the most significant leader features of ischemic brain injury mediated by acutely and chronically inflammation [68,69,70,71,72]. Syk is triggered by engaging molecules released by cells went forward necrosis [73,74,75] (Figure 2).

Once triggered, Syk (p-Syk) empowers the activation of a cascade constituted by different transcription factors such as PKB, p85, NF-κB, and PDK1 which codify proteins as COX-2, iNOS, and TNF-α; all molecules with pro-inflammatory proprieties [59,60,76,77,78,79].

In literature, various scientific researches have investigated the characteristic manifestations of neuroinflammation. Xin-Chun Ye et al. achieved this purpose examining the variation of concentrations of some inflammatory mediators such as Syk, Dectin-1, TNF-α, p-Syk, and inducible nitric oxide synthase (iNOS) in ischemic cerebral tissue of mice using in vitro and in vivo methods [15]. Tissue expression levels in damaged cerebral parenchyma after ischemia were found substantially augmented and they exert deleterious effects, enhancing the number of activated microglia and increasing neuroinflammation. Besides, they assessed that antagonizing the activity of Dectin-1 and Syk through respectively Dectin-1 antagonist laminarin (LAM) and Syk inhibitor piceatannol (PIC) results in a decreased synthesis of pro-inflammatory molecules levels and attenuated the number of activated microglia with a consequential reduction of neurological deficits. This finding could represent a novel therapeutic strategy capable of circumscribing ischemic damage, relieving organic and functional brain injury.

### 2.4. CXCL4-CCL5 Heterodimer

Macrophagic activities in the brain during a stroke are regulated by several inflammatory molecules, such as chemokines [80,81], small polypeptide categorized into four subsets like XC, CC, CX3C, and CXC [80,82]. CXCL4 is one of the most important of chemokines belonged to the category of the CXC subgroup because it has an impact on inflammation after an ischemic stroke [83,84,85,86], and CCL5 of the subgroup of CC modulates leukocyte migration. Besides, the relationship between CCL5 and its receptor CCR5, induces inflammatory response, cytophylaxis, and signal transcription [87].

Macrophages stem from brain resident microglia and circulating monocytes. A few hours after stroke onset, resident microglia and bloodstream monocytes are activated instantly and differentiate to macrophages. Microglia and monocyte-derived macrophages (indicated respectively with the acronyms MiMΦs and MoMΦs) do not differ from a morphological point of view should therefore be discernible identifying cell-surface markers through analyses which employ flow cytometric technique. Microglia-derived macrophages are CD45intCD11b+, instead monocyte-derived macrophages are CD45hiCD11b+. Furthermore, macrophages could be distinguished by additional markers such as CX3CR1, CCR2, and Ly6C.

During neuroinflammation, the capability of CCL5 of promoting monocyte adhesion and migration, consequentially to stimulation of CCR5, is empowered by establishment of the heterodimer.

The formation of CXCL4-CCL5 heterodimer could be void by a cyclic peptide synthesized on the base of CCL5 in mice called MKEY [16].

Evidence has shown that this inhibitor, preventing the assembly of the heterodimer mentioned above, is significantly capable of restrict the ischemic cerebral lesion and ameliorate neurologic deficits in mice, confirming once again the involvement of CXCL4-CCL5 in cerebral injury.

Besides, CCL5 and CCR5 could play a pivotal role in neuroinflammation in consideration of augmented expression of CCR5 on the surface of both subtypes of macrophages and increased levels of CCL5 in damaged parenchyma. In this way, the molecule, as mentioned above is responsible for the recruitment of white blood cells near the ischemic lesion [88], exacerbating inflammation, tissue detriment, and cerebral impairment [17]. Moreover, Yifang Fan et al. have also demonstrated that MKEY has the feature to suppress the activity of the MPO positive neutrophils, CD68-positive macrophages, and infiltrating monocytes-derived macrophages considerably, but not macrophages derived from microglia [89]. This assessment suggests that MKEY has a beneficial impact on the phenomenon of neuroinflammation right for the inhibition of monocytes-derived macrophages.

### 2.5. Microglial IRF5-IRF4 Regulatory Axis

Cerebral ischemic damage gets worse even for the role of the innate immune system because microglia could contribute to a secondary neuronal injury [90,91] and resident cerebral immune cells participate at ischemic pathophysiology, representing the main characters of the process of neuroinflammation [92].

Cuartero et al. supposed that neutrophils could assume a protective role in neuroinflammation after a stroke, shifting toward an N2 phenotype. They obtain this result by resorting to PPAR- γ, also known as peroxisome proliferator-activated receptor-γ, responsible for a mechanism of polarization. N2 neutrophils express M2 markers like CD206 that is a mannose receptor and Ym1 (also termed Chi3l3 or chitinase 3-like 3). They are related to increased neutrophil clearance, essential for resolving inflammation and neuroprotection. Besides, in literature, several studies postulate a significant relationship between neutrophils and endothelial cells [93]; indeed, cerebral endothelial cells after an ischemic event release CSF3 (Colony Stimulating Factor 3). This molecule determines the activation of neutrophil granulocytes, which, considering the dangerous activity of a scavenger receptor called CD36, causes toxic effects in damaged parenchyma.

Immune cells could acquire two different phenotypes “M1 and M2,” respectively, because they have two activation states after pathogenic stimulation that could lead or not to neuroinflammation, according to the inflammatory environment [92]. The first phenotype, M1, activated by realizing soluble FAS ligand, plays a part in ischemic lesion inducing the production of cytokines which enhanced inflammation and augmented infarct extent; the second phenotype M2, induced by IL-4, is characterized by neuroprotective properties, facilitating phagocytosis and enhancing neuroplasticity after stroke [94]. The reduction of microglia worsens postischemic inflammation, and brain damage in a study led on young mice [95,96], indicating that microglia are useful to reduce ischemic injury and are not merely neurotoxic.

Macrophage activation is mediated by interferon regulatory factors (IRF) in peripheral immune cells and other inflammatory diseases [18]. Recent studies have demonstrated that IRF5 and IRF4 expression in microglia exhibited a “see-saw” pattern and corresponded with pro-and anti-inflammatory profiles, respectively, after stroke [19]. These studies suggested the existence of an IRF5-IRF4 regulatory axis in which IRF5 signaling mediates microglial pro-inflammatory responses and IRF4 signaling enhances microglial anti-inflammatory activation, and as a result, the IRF5-IRF4 regulatory axis impacts stroke outcomes.

In contrast with other acute pathological changes (calcium overloading oxidative stress and others), the immune response to ischemic injury culminates later and lasts for months poststroke [97,98], offering us a more extended time window to do therapeutic acts to improve stroke outcomes potentially.

The microglial IRF5IRF4 regulatory axis can be potentially targeted to treat ischemic stroke and other neuroinflammatory diseases. Cell-specific therapy opens the door to new therapeutic possibilities represented by siRNA techniques and lentiviral vectors [99,100]. It constitutes a therapeutic line due to their capability to more precisely target cell-specific signaling pathways, eluding more global changes in immune regulation. Consequently, pharmacological reagents could be developed to reduce IRF5 or increase IRF4 expression in microglia after stroke, so that the pro-inflammatory response can be prevented and the anti-inflammatory response trigger earlier to clear the ischemic detriment and boost the tissue repair.

### 2.6. The Role of CD200-CD200R Interaction in Neuroinflammation after Stroke

In the scientific world, it is a well-established finding that neuroinflammation represents one of the prominent contributors to the pathogenetic mechanism of ischemic stroke, dramatically influencing the prognosis [101].

Therefore, different constituent elements belonged to the innate immune system are responsible for a protecting response against brain injury [102], through the release of mediators that promote brain regeneration. Moreover, several studies have also shown that after brain damage, other molecules may be responsible for brain impairment, hampering the process of neurogenesis and breaking the blood–brain barrier [9].

Hoek et al. demonstrated that after an ischemic stroke, neurons can enact a self-defense process across an interaction between CD200, an integral membrane glycoprotein member of immunoglobulin superfamily and CD200R, expressed on the microglial surface [103].

The inhibitory crosstalk realized between the ligand CD200 and its receptor called CD200R inhibitory immune ligand-receptor constitutes one of the most advantageous immunoregulatory systems that may avoid the release of pro-inflammatory cytokines in cerebral damage.

The loss of interplay between CD200-CD200R will induce microglial proliferation and activation that may exacerbate the process of neuroinflammation and aggravate the prognosis after stroke, as has been shown in a recent study conducted on CD200R1 knock-out mice, in which more deaths took place commensurate to the group of wild-type mice, probably because this induces monocyte infiltration and microgliosis [20].

Consequently, CD200 could play an important role in therapeutic strategies for the treatment of ischemic stroke by way of the inhibition of detrimental leukocyte activation and improvement of stroke-induced lymphopenia.

### 2.7. The Role of Astrocytes Activation in Neuroinflammation

Astrocytes are not only simple structural cells but are implicated in cerebral physiological and pathological processes such as neuroinflammation.

Evidence suggested that glial glutamate transporter (which acronym is GLT-1), an astrocytes’ transporter, after a brain ischemic event, could be responsible for opposite consequences because it takes up glutamate and consequently performing neuroprotective effects in the initial phases of ischemia. It is also capable of lengthening the ischemic damage because it issues glutamate and affects neuronal cells irreversibly until their death [21]. Astrogliosis is liable for changes in terms of morphology and function. Indeed, it determines the formation of a glial scar which has a neuroprotective role because it safeguards salubrious tissues from damages attributable to inflammation, allowing to restrict lesion size, but also may reduce infarct area recovery by diminish axons regeneration and preventing neuroplasticity [22]. Many molecules like p53 [104], p38 mitogen-activated protein kinase [105], acute-phase protein pentraxin-3 [106], and CD36 are implicated in astrocytes activation.

Consideration the assessments mentioned above, targeting astrocytes may represent an effective therapeutic strategy because, by extending livelihood of these cells, after an ischemic insult, it is possible to prolong neuronal continued-existence.

Indeed, Xu et al. demonstrated that astrocyte resistance is augmented determining an overexpression of superoxide dismutase-2 and HSP-72 (the acronym of heat shock protein 72). Through this mechanism, it is possible to preserve CA1 pyramidal neurons after a cerebrovascular accident involving the prosencephalon [107]. In the same way, pyruvate, increasing the production of glutathione, an antioxidant molecule which defends cells from toxicant agents like ROS, reduce injurious effects of glutamate activity in a heterogeneous culture of cortical cells [106,108]. Other studies have shown that p53 avoids impaired glutamate intake in astrocytes; consequently, it may be another therapeutic target in stroke lesion [104]. Some microRNAs expressed in astrocytes are also implicated in the physiopathologic mechanism of cerebral ischemia, therefore, by targeting them it is possible to ameliorate stroke outcome [109].

## 3. The Role of the Adaptative Immune System in Neuroinflammation

The term adaptative immunity (also known as acquired- or specific immunity) indicates an inflammatory response to pathological damage, and it includes some peculiar cell types called lymphocytes characterized by more effective and specific intervention, though at a slower pace, compared to the innate system against a pathogenic noxa.

The adaptive immune system could be dichotomously divided into humoral and cell-mediated immunity.

After a cerebrovascular accident, a finding of T cell activation and circulating antibodies supports the hypothesis that these exponents of acquired immunity could play a significant part in the inflammatory milieu related to brain injury.

Lymphocytes constitute one of the most important subgroups of white blood cells because they activate microglia, determinate a cerebral infarction of leukocytes, and influence the releasing of cytokines. Therefore, they have a crucial impact on stroke pathophysiologic mechanisms and the neuroinflammatory outcome. As known from many studies, several subtypes of lymphocytes such as TH17, γδ T-cells, and TH1 aggravate clinical prognosis, performing a pro-inflammatory activity, and the inhibition of their migration to the cerebral lesion allows to obtain better outcomes [110,111,112].

On the contrary, regulatory T-cells (also called Treg) have a neuroprotective role because they are capable of limiting disease [113]. The noxious effect of Treg depletion over the stroke size and outcomes have been investigated through a mechanism of cell lysis led by antibodies anti-CD25 [114]. Besides, for the same purpose, a study is conducted on transgenic mice with a receptor which binds the toxin of diphtheria (DTR) transgene. It is also used Foxp3 promoter for inducible Treg population reduction [115].

IL-33, Interleukin (IL)-2, T cell receptor recognition and serotonin promote the cerebral Treg cells proliferation, and the chemokines CCL1 and CCL20 are involved in the infiltration into the brain. Besides, as suggested by Ito et al. [116], in a recent experimental mice-model study, lymphocytes Treg in brain injury after stroke chronically regulate neurotoxic astrogliosis and neural rescue. They indeed release a ligand with low affinity for EGFR (the acronym of epidermal growth factor receptor) called amphiregulin.

Tuttolomondo et al. [117] analyzed the peripheral amount of a particular subcategory of T CD4+ lymphocytes which are defective for CD28 antigen in patients affected by ischemic brain injury considering the etiopathogenetic subset according to TOAST classification.

It has also been examined the relationship between CD28 null (also known as or CD4 + CD28- cells) with the entity of clinical severity of the cerebrovascular event valued through specific scores and with clinical outcomes comparatively diagnostic subset. It has been found that, during the acute phase of post-stroke neuroinflammation, CD4+ and CD28 null populations are more numerically represented and significantly predominate when the etiopathogenesis of stroke is cardioembolic compared to lacunar or LAAS subcategory. These results highlighted the current correlation between augmented clinical severity, larger dimension of brain lesions in patients affected by cardioembolic stroke and the intensity of the inflammatory response.

Nakajima et al. investigated the role of Killer immunoglobulin-like receptors, also named with acronym KIRs), in some pathologies sharing a common feature of atherosclerotic pathological burden [118].

These receptors are capable of identifying the target of T cells and NK cells through the interplay with HLA (the acronym of Human leukocyte antigen) class I molecules.

Taking into relationship the linkage between KIRs and atherosclerosis, newsworthy research is conducted by Tuttolomondo et al. in order to investigate a possible role in the etiopathogenesis of an acute cerebrovascular event [119].

Indeed, after a cerebral ischemic injury, it has been found an increased expression of KIR genes which enhances the process of inflammation related to augmented blood levels of CD28null.

The findings mentioned above corroborate the theory of the capability of KIR genes of modulating the expression of corresponding protein receptors, boosting through the release of several cytokines and the triggering of NK and T cells the complex phenomenon of neuroinflammation.

## 4. Relationship between Atrial Fibrillation and Neuroinflammation

Atrial fibrillation is defined as an irregular supraventricular tachyarrhythmia, and it can be diagnosed using electrocardiogram heart tracing. It determines in patients affected a fivefold chance of cerebral ischemia as compared with a normal population [120].

It represents one of the most common causes of cardioembolic stroke, and the chance that this inauspicious event occurs is equal in patients suffering from permanent AF and in people affected by paroxysmal AF, despite it, is frequently asymptomatic.

Therefore, concerning major risk to which people are suffering from the arrhythmia above-mentioned when its clinical presentation is without symptoms, it is absolutely necessary to discover a biomarker which can predict hopefully the probability that paroxysmal atrial fibrillation occurs.

Notwithstanding it has not yet be recognized as an index that meets all the requirements (high specific and sensible, inexpensive, high predictive, and rapid marker), several investigations are led on this issue.

Some of these studies have the purpose of molecules involved in the mechanism of fibrosis like MMP-9 (matrix metalloproteinase-9), Gal-3 (galectin 3), TGF-β, and PIIINP (the amino-terminal peptide of type III procollagen) [121,122,123].

Significant findings suggest an interesting correlation between the expression level of the molecules aforementioned and left atrial volume index (as known as LAVI), an effective predictor for the occurrence of atrial fibrillation.

Furthermore, serum concentrations of MMP-9, Gal-3 and PIIINP are found to be higher in people affected by atrial fibrillation, and that makes them possible upcoming predictors, in contrast to TGF-β with which it has not discovered a significative relationship with the arrhythmia concerned.

Other researches focused their attention on neurohormonal aspects in the context of atrial fibrillation, especially on atrial natriuretic peptides like NT-pro-BNP and BNP.

Interesting evidence of a study led by Naya et al. [124] indicates a relevant increment of levels of BNP and NTproBNP and a decrease of left atrial appendage flow in patients that undergo cardioembolic stroke. On this basis, these benchmarks could be helpful to distinguish cardioembolic from non-cardioembolic brain injury already from the first days.

Rodriguez-Yanez et al. recruited patients with cryptogenic stroke, and they have shown increased not only levels of NT-pro-BNP and BNP in these subjects but also a conjunction between this and higher significative chance to develop atrial fibrillation [125].

Up-to-date investigations have shown that, from a physiopathological point of view, cerebral cardioembolism is closely related to a condition of systemic inflammation [126].

Regarding possible inflammation markers, recent researches have described the usefulness of neutrophil-to-lymphocyte ratio (which acronym is NLR) in the role of a predictive marker of thromboembolic stroke in patients affected by atrial fibrillation [127,128].

A study lead by Nakase et al. has estimated the blood concentration of inflammatory mediators in the occasion of the acute phase of ischemic injury. In the present case, have been found elevated levels of hsCRP (high-sensitivity C-reactive protein), TNFα and IL-6, especially of the first molecule mentioned. Consequently, the hsCRP could be employed as an index of endothelial dysfunction in patients afflicted by atrial fibrillation [129].

In another research performed by Tuttolomondo et al. [130,131] on patients belonging to different pathological subcategories according to the classification TOAST, an increment of hematic levels of some inflammatory cytokines such as IL-1, TNFα, and IL-6 is observed in people members of CEI category compared to all the other ones.

Moreover, they expressed increased levels of vWF (von Willebrand factor) and more severe neurological impairment associated with an ominous prognosis.

All these scientific data found in literature denote the most severe entity of inflammation in patients with a cardioembolic stroke, and it is desirable leading further studies on this issue going forward to delineate new biomolecules which act as predictors of neuroinflammation, allowing to prevent subsequent brain injury.

## 5. Neuroinflammation and BBB Dysfunction

The blood–brain-barrier (also termed BBB) is defined as a membrane characterized by a refined selective permeability. Its complex structure is composed of different cell types including pericytes, proteins forming part of the extracellular matrix, endothelial cells, and astrocytes end-feet.

The cerebral homeostasis is maintained thanks to the selective transition of hematic substances through tight junctions between endothelial cells [132].

The rupture and the consequential dysfunction of BBB plays a crucial role in the process of neuroinflammation after an acute cerebrovascular accident, promoting poor prognosis. Indeed, the pathological passage of inflammatory mediators, toxins, and several cell types belonged to the immune system, provokes a major chance of hemorrhagic transformation of the infarctual lesion and an increment of vasogenic edema with resultant higher mortality [133].

Microglial can activate NADPH oxidase and as a result increasing the production of ROS, disrupting blood–brain-barrier [134] and mediates pro-cytokines’ secretion like interleukin (I.L.)1β and tumor necrosis factor (TNF-α) [135]. When ischemia provokes brain injury and BBB dysfunction, metalloproteinases, particularly MMP-2 and MMP-9 activated by microglia, degrade basal laminal components, and tight junctions [136], exacerbating focal cerebral ischemic lesion [137].

In order to investigate the biochemical mechanism that underlies the BBB rupture, different studies focused on the role of some miRNAs.

Indeed, among a variety of microRNAs are involved in pathophysiologic mechanisms such as angiogenesis, vascular inflammation, and atherosclerosis. Rom et al. identified let-7g and microRNA-98, two well-preserved miRNAs implicated in neuroinflammation since their expression is regulated by GSK3β inhibition [138].

GSK3β, also known as Glycogen synthase kinase 3β, is a multipurpose Serine/Threonine specific protein kinase identified in eukaryotic cells, which modulates the function of over 50 substrates through their phosphorylation. It takes part in plenty of fundamental cell activities, among other things, apoptosis, cell-cycle control, glycogen metabolism, cell differentiation, adhesion and motility, microtubule function, embryonic development, and inflammation [139]. It determines the impermeability of BBB in physiologic conditions, reduces the secretion of cytokines and other molecules involved in neuroinflammation in brain microvascular endothelial cells (BMVEC), has a safeguarding role against monocyte–endothelial interactions that induce BBB to break up and decreases monocyte adhesion to/migration across the BBB [140,141]. The Glycogen synthase kinase 3β is one of the factors involved in the endothelial dysfunction, that represents the first physiopathological step of vascular damage caused by inflammation [139,142]. The inhibition of GSK3β could play a prominent anti-inflammatory and BBB-protective role. Targeting GSK3β itself might provoke too side effects, as being involved in numerous physiological processes. Therefore, a solution can be represented by the regulation of miRNA expression.

In this study, Rom et al. suggested that elevated expression levels of the GSK3β inhibitor-dependent microRNAs, such as let-7g and miR-98, could prevent the adhesion of white blood cells and their migration across the blood–brain-barrier, whether with in vivo either with in vitro approaches [138]. These findings are very significant because not many microRNAs had equivalent capabilities to ameliorate the function of blood–brain-barrier in vitro. Indeed, micro-RNA let-7g markedly prevent the adhesion of leukocytes, but not their migration and its action are empowered by microRNA-98.

Two monocyte chemotactic proteins called RANTES/CCL5 and protein-1/CCL2 play a significant part in neuroinflammation since they rule the process of chemotaxis of white blood cells, and they allow them to cross the blood–brain-barrier [143]. Protein-1/CCL2 enhances small Rho GTPases, and this action determines the rearrangement of actin which constitutes the cytoskeleton and the formation of stress fiber. It also induces the reallocation of tight junction proteins such as occludin, claudin-5, ZO-1, and ZO-2 [144]. In this way, the monocyte chemotactic protein above mentioned is responsible for the modification of the tightness of blood–brain-barrier. To impede the release of RANTES/CCL5 and MCP-1/CCL2, cells of the endothelium are treated with GSK3β inhibitors [145]. Therefore, modulating the expression of let-7g and microRNA-98, it is possible to decrease the blood levels of the cytokines aforesaid and to neutralize the harmful consequences on proteins which constitute tight junctions and actin, likewise decreasing the interplay between endothelial cells and white cells.

Even though numerous researches have been carried out on physiopathogenetic mechanisms of BBB leakage, it is not entirely explained which molecules mediate this process in ischemic stroke.

In this regard, Liao et al., in an interesting study published in March 2020, analyzed the potential role of A-FABP, the acronym of adipocyte fatty acid-binding protein. It is an adipokine especially expressed in endothelial cells, adipocytes, and macrophages which exerts a proinflammatory action, determining metabolic dysfunction [146,147,148].

Previous studies have described increased serum levels of A-FABP in diabetic, obese, and hypertensive patients, therefore affected by factors of risk of a cerebral ischemic event [149] and they have also shown a link between these increased levels and an augmented serum expression of hsCRP [150], well-known inflammation index, which is increased in a cerebrovascular accident.

Liao et al. [151] experimented by recourse of knock-out and wild-type mice for expression of A-FABP gene submitted to a procedure of occlusion of the middle cerebral artery (MCAO). They described higher blood and intraparenchymal expression of this gene in wild-type mice. Besides, in knock-out mice a significative reduction of infarct size and entity of vasogenic edema with substantial alleviation of clinical neurological manifestations take place, suggesting a possible role of A-FABP in the pathogenesis of ischemic brain injury. Indeed, in mice with higher levels of this adipokine, they observed enhanced transcription of MMP-9 by resident microglia, and peripheral macrophages through the activation of JNK-Jun pathway, responsible for the deterioration of tight junctions and subsequential BBB leakage.

Moreover, the genetic and pharmacological modulation of A-FABP could represent a promising therapeutic strategy, inhibiting its action of BBB demolition related to MMP-9 hyperactivation, and, consequently, relieving ischemic damage.

## 6. miRNAs and Neuroinflammation

In the last decenniums, it has been identified as a new subset of small non-coding RNAs called microRNAs or miRs. They are short sequences of 20–24 nucleotides of single-stranded non-coding RNA implicated in the regulation of endothelial dysfunction and cancer angiogenesis, silencing the expressions of the genes involved in these processes [152], but also in inflammatory, metabolic, neurodegenerative, and immunity phenomena.

Since it is possible to seek out microRNAs in different human biologic fluids like the serum, plasma, cerebrospinal fluid, and urine, numerous studies have the purpose to evaluating possible role as diagnostic and therapeutic markers of this sequences in different pathologies, including ischemic stroke [153].

These studies have shown that the expression miRNA-126, miRNA-124-3p, miRNA30a, and miRNA16 e let-7b are considerably elevated in patients with an acute ischemic brain lesion, but they are not employed with success in clinical routine [153,154].

In pioneering research led by Wang et al. in patients suffering from ischemic stroke, were discovered targets of miRNA382-5p, miRNA 221-3p, and miRNA-4271. They are respectively DNA topoisomerase I (TOP1; it modulates and modifies DNA strands during transcription), alpha-receptor of GABA (GABRA 1; which transmits cerebral inhibitory cues) and a beta-containing ubiquitin-protein ligase (BTRC; it is responsible for the demolition of CD4 after the interplay between HIV-1p). Other studies have demonstrated that miRNA-382-5p has the target of NFIA, the acronym of nuclear factor IA which is implied in the modulation of cholesterol metabolism and inflammatory response [21]; PGC-1a as known as peroxisome proliferator-activated receptor γ coactivator 1a, that mediates the pathogenetic process of atherosclerosis [155]. Besides, they described APOC3, also called apolipoprotein C-III, which is a direct target of mRNA-4271 that regulates serum concentrations of triglycerides [156].

Therefore, these miRNAs have all targets involved in etiopathogenetic substrates of ischemic injury.

A further study published in 2017, Wang et al. have revealed that in subjects suffered a stroke compared to normal people, the serum expression of miRNA-382-5p and miRNA-221-3p are significantly reduced, proposing a possible role in the quality of non-invasive diagnostic marker of stroke [157].

Other researches have suggested a relationship between the microRNA-3845p and various disease which share the same pathologic substrate of ischemia like atherosclerosis and ischemia of myocardium [158,159]. Additionally, a current investigation has shown that expression levels of microRNA-384-5p are lower in murine cells of ischemic myocardium and ventricular cells in hypoxic conditions [160]. Furthermore, considering the role of specific microRNAs in the regulation of ischemic disease, they could open a new health direction to improve prophylaxis and modify clinical outcomes after an ischemic brain injury [161]. Indeed, some studies have reported that miR-137 safeguards neurons against impairment related to reperfusion and ischemia modulating the Notch signaling cascade [162]. The Notch receptor engages itself to DLL4, the acronym of Delta-like ligand 4. When the expression level of this ligand is elevated, the Notch signaling cascade is activated, and this impedes the sprouting of endothelial cells during the growth of blood vessels [163].

Several determinants like molecular modulators in the initial phase of embryogenesis, Notch receptors and downstream signaling patterns influence the regulation of the angiogenesis process by the Notch signaling pathway.

Different molecules mediate the process of angiogenesis. Among all of them, DLL4, a Notch ligand with high specificity for this receptor of endothelial progenitor cells, has a significant impact on vessel development [164,165]. Jia Fan et al., in an up-to-date analysis about microRNA-384-5p supposed that it could be involved in the pathogenesis of cerebral ischemic event because it influences the activation of DDL4 and, consequently, the process of angiogenesis [145].

They showed that the therapeutic use of microRNA-3845p mimics and small interference RNA Delta-like ligand 4 (also known as si-DLL4) in mice in which is realized a middle cerebral artery occlusion, reduce the extension of lesion, decreasing the populations of necrotic and karyopyknotic the number of and necrotic cortical cells, and is responsible for lower expression levels of VEGF protein and CD31. Therefore, other researches have underlined that another small non-coding-RNA called miRNA-181b can prevent the cleavage of caspase-3, reducing the entity of loss of neurons in the cerebral ischemic cortex, by attenuating neurological loss of function of mice after the realization of middle cerebral artery occlusion [166].

Moreover, microRNA-384-5p may constitute an innovative approach for the therapy of cerebral ischemic stroke because it modulates angiogenesis and proliferation of endothelial progenitor cells and prevents neuronal apoptosis from interacting with DDL4 in the Notch signaling pathway.

## 7. Conclusions

Brain ischemic injury represents the second most frequent reason for death and long term disablement throughout the world. From the etiopathogenic point of view, TOAST classification has described several stroke subtypes. They could be large-artery atherosclerosis, cardioembolism, systemic hypoperfusion, penetrating artery disease, carotid dissection, hypercoagulability of genetic syndromes or with an undetermined etiology. By which, atherosclerosis formation has been blamed to be one of the culprits for the inclining cases of stroke-related death.

Therefore, atherosclerosis acts like the trigger of an intricate chain of events which modify molecular, genomic and cellular frameworks and inflammation has a pivotal role in this cascade, both in the central and in the peripheric nervous system. After an ischemic stroke, several cytokines take part in neuroinflammation, and their actions have repercussions on increasing infarct size, brain impairment, and clinical prognosis. Clinical findings have evidence that, in the acute phase, it is essential preventing deleterious effects of mediators of neuroinflammation to avoid poor outcomes.

Considering these assessments, in recent years, many authors have investigated the role of several molecules involved in atherosclerosis to identify innovatory biomarkers which are the expression of worsening outcomes. In this way, patients who have more substantial death and disability risk may be subject to frequent and accurate monitoring and appropriate therapeutic protocols.

Moreover, it could be auspicious that future studies will contribute to the detection and the comprehending of an increasing number of genetic, biological, and molecular key factors of stroke, in term of predisposition to the disease, establishment, and progression of the damage. The results of these studies could, in future, lay the cornerstone for identification of new therapeutic targets that may allow a significant modification of death and disability rate.

## Figures and Tables

**Table 1:** Key biological molecular patterns of innate immune system implied in neuroinflammation.

Evidence	Findings	References
NLRP3 inflammasome is one of the main contributors to the process of neuroinflammation and consequent brain damage.	NLRP3 inflammasome is responsible for activation of IL-1β, and the release of IL-18, which phosphorylate IRS-1, worsening insulin-resistance and causing neuronal death.	[11,12]
High levels or low levels of DKK-3 worsen outcome after an ischemic stroke.	DKK-3 concentrations have an impact on endothelial dysfunction and atherosclerosis.	[13,14]
The inflammatory pathway mediated by Dectin-1/Syk has a pivotal role in neuroinflammation after a stroke.	The crosstalk between Dectin-1 and DAMPs determines phosphorylation of ITAM and, subsequently, of Syk, a kinase which mediates a cascade of neuroinflammation and the release of several cytokines.	[15]
The heterodimer CXCL4-CCL5 plays a crucial part in the cerebral injury.	Avoiding the formation of CXCL4-CCL5 heterodimer, MKEY, a cyclic peptide synthesized on the base of CCL5 in mice, restricts the ischemic cerebral lesion and ameliorate neurologic deficits in mice.	[16,17]
The microglial IRF5-IRF4 regulatory axis impacts stroke outcomes.	Studies suggested the existence of an IRF5-IRF4regulatory axis in which IRF5 signaling mediates microglial pro-inflammatory responses and IRF4 signaling enhances microglial anti-inflammatory activation.	[18,19]
CD200 could play an important role in therapeutic strategies for the treatment of ischemic stroke by way of the inhibition of detrimental leukocyte activation and improvement of stroke-induced lymphopenia.	The loss of interplay between CD200-CD200R will induce microglial proliferation and activation that may exacerbate the process of neuroinflammation and aggravate the prognosis after stroke.	[20]
The glial glutamate transporter (GLT-1), an astrocytes’ transporter, performs neuroprotective effects in the initial phases of ischemia.	It determines the formation of a glial scar which has a neuroprotective role because it safeguards salubrious tissues from damages attributable to inflammation.	[21,22]

## Abbreviations

A-FABP	Adipocyte fatty acid-binding protein
AF	Atrial fibrillation
ApoE	Apolipoprotein E
ApoC3	Apolipoprotein C-III
ASC	Apoptosis-associated speck-like protein containing a CARD
BBB	Blood–brain barrier
BTRC	Beta-transducin repeat containing
CCL1	Chemokine (C-C motif) ligand 1
CCL5	Chemokine (C-C motif) ligand 5
CCL20	Chemokine (C-C motif) ligand 20
CCR5	C-C chemokine receptor type 5
CLEC2	C-type lectin-like receptor 2
CLEC9A	C-type lectin-like receptor 9A
COX-2	Cyclooxygenase-2
CSF-3	Colony-stimulating factor 3
CX3CL1	Chemokine (C-X3-C motif) ligand 1
CXCL4	Chemokine (C-X-C motif) ligand 4
CXCL7	Chemokine (C-X-C motif) ligand 7
CXCL8	Chemokine (C-X-C motif) ligand 8
DAMPs	Damage-associated molecular patterns
DLL-4	Delta-like ligand 4
DKK-3	Dickkopf WNT Signaling Pathway Inhibitor 3
FOXP3	Forkhead box P3
GABRA 1	Alpha receptor GABA 1
Gal-3	Galectin-3
GLT-1	Glial glutamate transporter
GSK3 β	Glycogen synthase kinase 3 beta
hemITAMs	Hemi-immunoreceptor tyrosine-based activation motifs
hsCRP	High-sensitivity C-reactive protein
HSP-72	Heat shock protein 72
IL-1α	Interleukin 1 alpha
IL-1β	Interleukin 1 beta
IL-6	Interleukin
IL-7	Interleukin 7
IL-10	Interleukin 10
IL-17	Interleukin 17
IL-18	Interleukin 18
IL-20	Interleukin 20
IL-23	Interleukin 23
iNOS	Inducible nitric oxide synthase
IRF4	Interferon regulatory factor 4
IRF5	Interferon regulatory factor 5
IRS-1	Insulin receptor substrate 1
ITAMs	Immunoreceptor tyrosine-based activation motifs
JNK	c-Jun N-terminal kinase
KIRs	Killer immunoglobulin-like receptors
LAM	Laminarin
LRR	Leucine-rich repeats
Ly6C	Ly6Chi monocytes
MCAO	Middle cerebral artery occlusion
MiMΦs	Microglia-derived macrophages
miRNAs	MicroRNAs
MoMΦs	Monocyte-derived macrophages
MPO	Myeloperoxidase
MMP-9	Matrix metalloproteinase 9
NADPH oxidase	Nicotinamide adenine dinucleotide phosphate oxidase
NAIP	Neuronal apoptosis inhibitory protein
NLR	NOD-like receptor
NLR	Neutrophil-to-lymphocyte ratio
NLRP3	NLR family pyrin domain containing 3
NFIA	Nuclear factor IA
PIC	Piceatannol
PIIINP	Amino-terminal peptide of type III procollagen
PGC-1a	Peroxisome proliferator-activated receptor γ coactivator 1
PPAR-γ	Peroxisome proliferator-activated receptor gamma
PYCARD	PYD And CARD Domain Containing
PYD	Pyrin domain
ROS	Reactive oxygen species
siRNA	Small interfering RNA
SYK	Spleen tyrosine kinase
TGF-β	Transforming growth factor-beta
TNF-α	Tumor Necrosis Factor-alpha
TOP1	DNA topoisomerase I
Tregs	Regulatory T cells
TXNIP	Thioredoxin-interacting protein
VDUP-1	Vitamin D3-upregulated protein-1
VEGF	Vascular endothelial growth factor
vWF	von Willebrand factor
Wnt	Wingless-related integration site
